# Creatinium perchlorate

**DOI:** 10.1107/S1600536809003171

**Published:** 2009-02-04

**Authors:** Amel Messai, Amani Direm, Nourredine Benali-Cherif, Dominique Luneau, Erwann Jeanneau

**Affiliations:** a Laboratoire des Structures, Propriétés et Interactions Interatomiques, Centre Universitaire de Khenchela, 40000 Khenchela, Algeria; b Laboratoire des Multimatériaux et Interfaces, UMR 5615, Université Claude Bernard Lyon1, 69622 Villeurbanne Cedex, France

## Abstract

The title compound, C_4_H_8_N_3_O^+^·ClO_4_^−^, is built up from creatininium cations and perchlorate anions. Crystal cohesion and perchlorate stability are ensured by N—H⋯O hydrogen bonds that together with weak C—H⋯O inter­actions build up a three-dimensional network.

## Related literature

For background on organic–inorganic hybrid materials, see: Benali-Cherif *et al.* (2004 ▶); Hill (1998 ▶); Kagan *et al.* (1999 ▶). For a related structure, see: Cherouana *et al.* (2003 ▶); Berrah *et al.* (2005 ▶). For inter­pretation of the solution acidity effect on NMR chemical shifts, see: Kotsyubynskyy *et al.* (2004 ▶). 
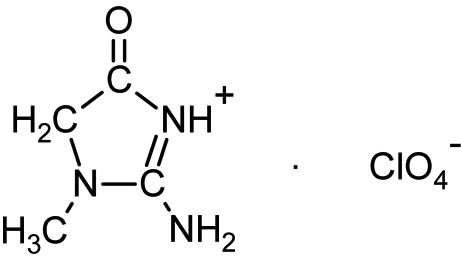


## Experimental

### 

#### Crystal data

C_4_H_8_N_3_O^+^·ClO_4_^−^
*M* *_r_* = 213.58
Monoclinic, 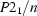

*a* = 5.8023 (3) Å
*b* = 20.7782 (13) Å
*c* = 7.3250 (4) Å
β = 107.947 (4)°
*V* = 840.14 (8) Å^3^
*Z* = 4
Mo *K*α radiation
μ = 0.45 mm^−1^
*T* = 293 (2) K
0.10 × 0.10 × 0.10 mm

#### Data collection

Nonius KappaCCD diffractometer
Absorption correction: none
4080 measured reflections
1587 independent reflections
1209 reflections with *I* > 2σ(*I*)
*R*_int_ = 0.126

#### Refinement

*R*[*F* ^2^ > 2σ(*F* ^2^)] = 0.075
*wR*(*F* ^2^) = 0.230
*S* = 1.05
1587 reflections
119 parameters
H-atom parameters constrained
Δρ_max_ = 0.39 e Å^−3^
Δρ_min_ = −0.42 e Å^−3^

### 

Data collection: *COLLECT* (Nonius, 1998 ▶); cell refinement: *SCALEPACK* (Otwinowski & Minor, 1997 ▶); data reduction: *SCALEPACK* and *DENZO* (Otwinowski & Minor, 1997 ▶); program(s) used to solve structure: *SIR92* (Altomare *et al.*, 1993 ▶); program(s) used to refine structure: *SHELXL97* (Sheldrick, 2008 ▶); molecular graphics: *ORTEP-3 for Windows* (Farrugia, 1997 ▶) and *PLATON* (Spek, 2003 ▶); software used to prepare material for publication: *WinGX* (Farrugia, 1999 ▶).

## Supplementary Material

Crystal structure: contains datablocks global, I. DOI: 10.1107/S1600536809003171/dn2425sup1.cif

Structure factors: contains datablocks I. DOI: 10.1107/S1600536809003171/dn2425Isup2.hkl

Additional supplementary materials:  crystallographic information; 3D view; checkCIF report

## Figures and Tables

**Table 1:** Hydrogen-bond geometry (Å, °)

*D*—H⋯*A*	*D*—H	H⋯*A*	*D*⋯*A*	*D*—H⋯*A*
N1—H1⋯O4	0.86	2.18	2.905 (5)	142
N2—H2*B*⋯O4	0.86	2.33	3.044 (6)	141
N2—H2*A*⋯O2^i^	0.86	2.31	3.077 (6)	148
N2—H2*A*⋯O2^ii^	0.86	2.52	3.186 (5)	136
N2—H2*B*⋯O3^iii^	0.86	2.39	2.947 (5)	123
C3—H3*A*⋯O2^ii^	0.96	2.51	3.455 (5)	168
C4—H4*A*⋯O1^iv^	0.97	2.43	3.284 (5)	147

Symmetry codes: (i) 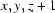; (ii) 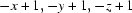; (iii) 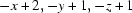; (iv) 
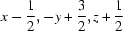
.

**Fig. 1.**
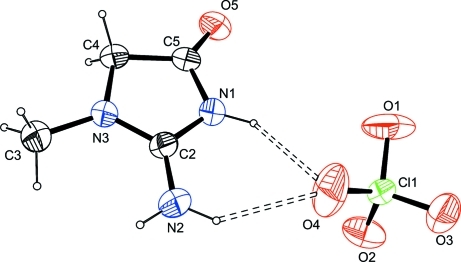
Asymmetric unit and atom-numbering scheme of creatininium perchlorate. Displacement ellipsoids are drawn at the 30% probability level. H atoms are represented as small sphere of arbitrary radii. H bonds are shown as dashed lines.

**Fig. 2.**
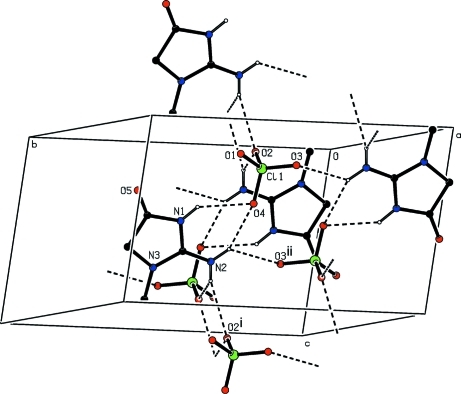
Partial packing view showing the hydrogen bond pattern between cation and anion. H atoms not involved in hydrogen bonding have been omitted for clarity. [Symmetry codes: (i) x, y, z+1; (ii) 2-x, 1-y, 1-z]

## supplementary crystallographic information

### Comment

Studies of organic-inorganic hybrid materials, including amino acids and various inorganic acids (Benali-Cherif *et al.*, 2004), have received a great deal of attention in recent years, because of their electrical, magnetic and optical properties (Kagan *et al.*, 1999; Hill, 1998).

Creatinine is formed by the metabolism of phosphocreatine, a high-energy molecule which provides a rapid supply of ATP to muscles. Phosphocreatine is converted spontaneously to creatinine on a regular basis. Consequently, creatinine is released into the blood and excreted by the kidneys as a metabolic waste product.

The present structure analysis of creatininium perchlorate, (I), was undertaken as part of a more general investigation into the nature of hydrogen bonding between organic bases or amino acids and inorganic acids in their crystalline forms (Cherouana *et al.*, 2003).

In the present study, only the imino group of the imidazolyl moiety (atom N1) in creatinine is protonated, which confirms the possibility of the existence of creatininium cations in various tautomeric forms in aqueous solution. This is discussed and quantified in the light of the interpretation of the solution acidity effect on 1H, 13 C and 14 N NMR chemical shifts (Kotsyubynskyy *et al.*, 2004).

The asymmetric unit of (I) contains a monoprotonated creatininium cation and two perchlorate anions (Fig.1).

The bond distances in the imidazolyl ring of (I) are, in general, not significantly different from those found in similar hybrid compounds containing protonated imidazolyl moieties like creatininium nitrate (Berrah *et al.*, 2005). The creatininium ring is planar, as expected, with a mean deviation from planarity of 0.0017 Å.

The average Cl—O bond distances and O—Cl—O bond angles are 1.40625 (4) Å and 109.50 (3)°, respectively, confirming a tetrahedral configuration, similar to other perchlorates studied at low temperature. Perchlorate anions (ClO_4_^-^), surrounded by two creatininium residues *via* hydrogen bonds play an important role in stabilizing the crystal structure.

The cation-anion N—H—O interactions form sheet parallel to the (0 1 0) plane (Table 1, Fig.2). Weak C-H···O interactions further link the sheets to form a three dimensionnal network (Table 1).

### Experimental

The title compound (I) was cristallized by slow evaporation at room temperature of an aqueous solution of creatinine and perchloric acid in a 1:1 stochiometric ratio.

### Refinement

The title compound crystallizes in the centrosymmetric space group *P*2_1_/n. All non-H atoms were refined with anisotropic atomic displacement parameters. All H-atoms of the cation entities were located in difference Fourier syntheses and refined as riding model with C—H and N—H bond lengths constrained to 0.96–0.97 Å and 0.834 Å, respectively.

### Crystal data

**Table:**

C_4_H_8_N_3_O^+^·ClO_4_^−^	*F*(000) = 440
*M**_r_* = 213.58	*D*_x_ = 1.689 Mg m^−^^3^
Monoclinic, *P*2_1_/*n*	Mo *K*α radiation, λ = 0.71073 Å
Hall symbol: -P 2yn	Cell parameters from 4863 reflections
*a* = 5.8023 (3) Å	θ = 2.0–26.0°
*b* = 20.7782 (13) Å	µ = 0.45 mm^−^^1^
*c* = 7.3250 (4) Å	*T* = 293 K
β = 107.947 (4)°	Prism, colourless
*V* = 840.14 (8) Å^3^	0.10 × 0.10 × 0.10 mm
*Z* = 4	

### Data collection

**Table:**

Nonius KappaCCD diffractometer	1209 reflections with *I* > 2σ(*I*)
Radiation source: fine-focus sealed tube	*R*_int_ = 0.126
graphite	θ_max_ = 26.0°, θ_min_ = 2.0°
ω–θ scans	*h* = −7→6
4080 measured reflections	*k* = −23→25
1587 independent reflections	*l* = −9→9

### Refinement

**Table:**

Refinement on *F*^2^	Primary atom site location: structure-invariant direct methods
Least-squares matrix: full	Secondary atom site location: difference Fourier map
*R*[*F*^2^ > 2σ(*F*^2^)] = 0.075	Hydrogen site location: inferred from neighbouring sites
*wR*(*F*^2^) = 0.230	H-atom parameters constrained
*S* = 1.05	*w* = 1/[σ^2^(*F*_o_^2^) + (0.139*P*)^2^ + 0.288*P*] where *P* = (*F*_o_^2^ + 2*F*_c_^2^)/3
1586 reflections	(Δ/σ)_max_ = 0.003
119 parameters	Δρ_max_ = 0.39 e Å^−^^3^
0 restraints	Δρ_min_ = −0.41 e Å^−^^3^

### Special details

**Table:**

Geometry. All e.s.d.'s (except the e.s.d. in the dihedral angle between two l.s. planes) are estimated using the full covariance matrix. The cell e.s.d.'s are taken into account individually in the estimation of e.s.d.'s in distances, angles and torsion angles; correlations between e.s.d.'s in cell parameters are only used when they are defined by crystal symmetry. An approximate (isotropic) treatment of cell e.s.d.'s is used for estimating e.s.d.'s involving l.s. planes.
Refinement. Refinement of *F*^2^ against ALL reflections. The weighted *R*-factor *wR* and goodness of fit *S* are based on *F*^2^, conventional *R*-factors *R* are based on *F*, with *F* set to zero for negative *F*^2^. The threshold expression of *F*^2^ > σ(*F*^2^) is used only for calculating *R*-factors(gt) *etc*. and is not relevant to the choice of reflections for refinement. *R*-factors based on *F*^2^ are statistically about twice as large as those based on *F*, and *R*- factors based on ALL data will be even larger.

### Fractional atomic coordinates and isotropic or equivalent isotropic displacement parameters (Å^2^)

**Table:**

	*x*	*y*	*z*	*U*_iso_*/*U*_eq_	
O5	0.3264 (6)	0.74385 (13)	0.3068 (5)	0.0803 (9)	
N1	0.4969 (5)	0.65462 (13)	0.4848 (5)	0.0623 (8)	
H1	0.5899	0.6421	0.4202	0.075*	
N2	0.5866 (6)	0.57013 (14)	0.7078 (6)	0.0723 (10)	
H2A	0.5667	0.5522	0.8077	0.087*	
H2B	0.6800	0.5526	0.6508	0.087*	
N3	0.3285 (5)	0.65583 (13)	0.7149 (4)	0.0577 (8)	
C2	0.4753 (6)	0.62392 (16)	0.6428 (5)	0.0555 (8)	
C3	0.2475 (8)	0.6361 (2)	0.8757 (6)	0.0729 (11)	
H3A	0.2690	0.5905	0.8942	0.109*	
H3B	0.0793	0.6466	0.8491	0.109*	
H3C	0.3409	0.6581	0.9897	0.109*	
C4	0.2340 (7)	0.71236 (15)	0.5973 (6)	0.0622 (10)	
H4A	0.2797	0.7516	0.6713	0.075*	
H4B	0.0588	0.7106	0.5451	0.075*	
C5	0.3498 (7)	0.70880 (15)	0.4411 (6)	0.0628 (10)	
Cl1	0.89960 (16)	0.57125 (4)	0.24596 (14)	0.0599 (5)	
O1	0.8635 (8)	0.63394 (16)	0.1663 (8)	0.1392 (18)	
O2	0.7200 (7)	0.52990 (16)	0.1320 (6)	0.1017 (12)	
O3	1.1298 (6)	0.5496 (2)	0.2543 (6)	0.1112 (13)	
O4	0.8863 (10)	0.5762 (3)	0.4326 (7)	0.1394 (19)	

### Atomic displacement parameters (Å^2^)

**Table:**

	*U*^11^	*U*^22^	*U*^33^	*U*^12^	*U*^13^	*U*^23^
O5	0.105 (2)	0.0604 (16)	0.085 (2)	−0.0036 (14)	0.0420 (17)	0.0152 (14)
N1	0.0699 (17)	0.0527 (16)	0.075 (2)	−0.0020 (13)	0.0384 (16)	−0.0039 (14)
N2	0.082 (2)	0.0549 (19)	0.088 (2)	0.0147 (14)	0.0381 (19)	0.0059 (15)
N3	0.0663 (16)	0.0474 (15)	0.0692 (19)	0.0027 (13)	0.0353 (14)	0.0031 (13)
C2	0.0587 (18)	0.0449 (16)	0.066 (2)	−0.0009 (14)	0.0244 (16)	−0.0049 (15)
C3	0.087 (3)	0.064 (2)	0.079 (3)	0.008 (2)	0.042 (2)	0.0080 (19)
C4	0.074 (2)	0.0415 (17)	0.081 (3)	0.0048 (15)	0.0369 (19)	0.0024 (15)
C5	0.072 (2)	0.0421 (17)	0.081 (3)	−0.0083 (15)	0.0334 (19)	−0.0017 (16)
Cl1	0.0651 (7)	0.0471 (6)	0.0728 (7)	0.0028 (3)	0.0289 (5)	−0.0067 (3)
O1	0.144 (3)	0.0463 (18)	0.198 (5)	0.0036 (19)	0.010 (3)	0.017 (2)
O2	0.110 (2)	0.074 (2)	0.108 (3)	−0.0300 (17)	0.014 (2)	0.0009 (17)
O3	0.088 (2)	0.104 (3)	0.155 (4)	0.015 (2)	0.058 (2)	−0.016 (3)
O4	0.164 (4)	0.187 (5)	0.091 (3)	0.029 (3)	0.074 (3)	−0.019 (3)

### Geometric parameters (Å, °)

**Table:**

O5—C5	1.197 (5)	C3—H3A	0.9600
N1—C2	1.361 (4)	C3—H3B	0.9600
N1—C5	1.389 (5)	C3—H3C	0.9600
N1—H1	0.8600	C4—C5	1.497 (6)
N2—C2	1.305 (4)	C4—H4A	0.9700
N2—H2A	0.8600	C4—H4B	0.9700
N2—H2B	0.8600	Cl1—O3	1.393 (3)
N3—C2	1.312 (4)	Cl1—O4	1.396 (4)
N3—C3	1.455 (5)	Cl1—O2	1.409 (3)
N3—C4	1.460 (4)	Cl1—O1	1.416 (4)
			
C2—N1—C5	111.4 (3)	H3B—C3—H3C	109.5
C2—N1—H1	124.3	N3—C4—C5	103.6 (3)
C5—N1—H1	124.3	N3—C4—H4A	111.0
C2—N2—H2A	120.0	C5—C4—H4A	111.0
C2—N2—H2B	120.0	N3—C4—H4B	111.0
H2A—N2—H2B	120.0	C5—C4—H4B	111.0
C2—N3—C3	126.5 (3)	H4A—C4—H4B	109.0
C2—N3—C4	110.0 (3)	O5—C5—N1	126.0 (4)
C3—N3—C4	123.3 (3)	O5—C5—C4	129.3 (3)
N2—C2—N3	126.6 (4)	N1—C5—C4	104.6 (3)
N2—C2—N1	123.1 (3)	O3—Cl1—O4	108.8 (3)
N3—C2—N1	110.3 (3)	O3—Cl1—O2	110.7 (3)
N3—C3—H3A	109.5	O4—Cl1—O2	111.8 (3)
N3—C3—H3B	109.5	O3—Cl1—O1	109.5 (3)
H3A—C3—H3B	109.5	O4—Cl1—O1	106.8 (3)
N3—C3—H3C	109.5	O2—Cl1—O1	109.2 (2)
H3A—C3—H3C	109.5		

### Hydrogen-bond geometry (Å, °)

**Table:**

*D*—H···*A*	*D*—H	H···*A*	*D*···*A*	*D*—H···*A*
N1—H1···O4	0.86	2.18	2.905 (5)	142
N2—H2B···O4	0.86	2.33	3.044 (6)	141
N2—H2A···O2^i^	0.86	2.31	3.077 (6)	148
N2—H2A···O2^ii^	0.86	2.52	3.186 (5)	136
N2—H2B···O3^iii^	0.86	2.39	2.947 (5)	123
C3—H3A···O2^ii^	0.96	2.51	3.455 (5)	168
C4—H4A···O1^iv^	0.97	2.43	3.284 (5)	147

Symmetry codes: (i) *x*, *y*, *z*+1; (ii) −*x*+1, −*y*+1, −*z*+1; (iii) −*x*+2, −*y*+1, −*z*+1; (iv) *x*−1/2, −*y*+3/2, *z*+1/2.
